# Supplementary material for: Conditional chemoconnectomics (cCCTomics) as a strategy for efficient and conditional targeting of chemical transmission
Source: eLife. 2024 Apr 30;12:RP91927. doi: 10.7554/eLife.91927 (PMC11060718; doi:10.7554/eLife.91927)
Supplement: Figure 3—source data 3. [file elife-91927-fig3-data3.zip › Figure 3 source data3 part 1.zip!/Figure 3-Source data 3-part1/1071-1-CRISPResso_Report_osfbjvga.zip!/CRISPResso_on_1071_4/1c.Alignment_barplot.pdf]

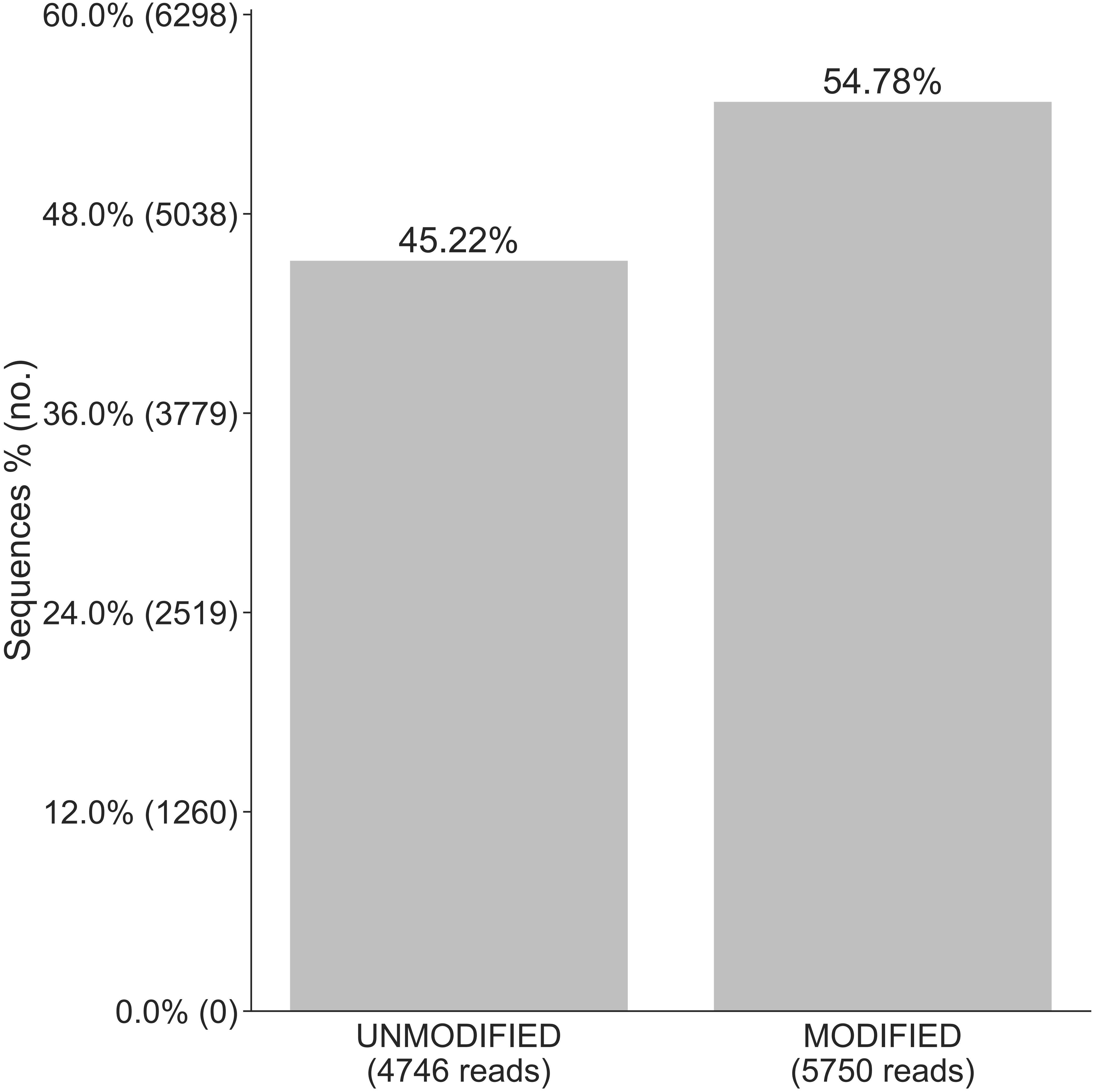
60.0% (6298)
48.0% (5038)
36.0% (3779)
24.0% (2519)
12.0% (1260)
0.0% (0)
Sequences % (no.)
45.22%
54.78%
UNMODIFIED
(4746 reads)
MODIFIED
(5750 reads)
